# Axotrophin/MARCH7 acts as an E3 ubiquitin ligase and ubiquitinates tau protein *in vitro* impairing microtubule binding

**DOI:** 10.1016/j.bbadis.2014.05.029

**Published:** 2014-09

**Authors:** Katharina Flach, Ellen Ramminger, Isabel Hilbrich, Annika Arsalan-Werner, Franziska Albrecht, Lydia Herrmann, Michel Goedert, Thomas Arendt, Max Holzer

**Affiliations:** a Paul Flechsig Institute of Brain Research, Department of Molecular and Cellular Mechanisms of Neurodegeneration, University of Leipzig, 04109 Leipzig, Germany; b MRC, Laboratory of Molecular Biology, Neurobiology Division, Francis Crick Avenue, Cambridge CB2 0QH, UK

**Keywords:** Alzheimer's disease, Tau protein, Mono-ubiquitination, Ring-variant domain, Yeast two-hybrid assay, Microtubule binding

## Abstract

Tau is the major microtubule-associated protein in neurons involved in microtubule stabilization in the axonal compartment. Changes in tau gene expression, alternative splicing and posttranslational modification regulate tau function and in tauopathies can result in tau mislocalization and dysfunction, causing tau aggregation and cell death. To uncover proteins involved in the development of tauopathies, a yeast two-hybrid system was used to screen for tau-interacting proteins. We show that axotrophin/MARCH7, a RING-variant domain containing protein with similarity to E3 ubiquitin ligases interacts with tau. We defined the tau binding domain to amino acids 552–682 of axotrophin comprising the RING-variant domain. Co-immunoprecipitation and co-localization confirmed the specificity of the interaction. Intracellular localization of axotrophin is determined by an N-terminal nuclear targeting signal and a C-terminal nuclear export signal. In AD brain nuclear localization is lost and axotrophin is rather associated with neurofibrillary tangles. We find here that tau becomes mono-ubiquitinated by recombinant tau-interacting RING-variant domain, which diminishes its microtubule-binding. In vitro ubiquitination of four-repeat tau results in incorporation of up to four ubiquitin molecules compared to two molecules in three-repeat tau. In summary, we present a novel tau modification occurring preferentially on 4-repeat tau protein which modifies microtubule-binding and may impact on the pathogenesis of tauopathies.

## Highlights

• We search for tau-interacting proteins using a cytotrap yeast two-hybrid assay.
• MARCH7 was identified as a tau-binding protein and confirmed by several methods.
• Recombinant MARCH7 Ring-variant domain uses Ubc5 for E3 self-ubiquitinating activity.
• MARCH7 Ring-variant domain mono-ubiquitinates tau protein at multiple sites including the microtubule-binding domain.
• Mono-ubiquitination of tau protein diminishes its microtubule-binding.

## 1 Introduction

Tau protein belongs to the family of microtubule-associated proteins (MAPs) and is predominantly expressed in neurons, where it is enriched in axons. Tau protein binds to microtubules and promotes their assembly and stabilization. Different tau isoforms generated by alternative splicing display variable microtubule binding capacities. In the human brain six isoforms of tau protein, that arise from a single gene by alternative mRNA splicing of exons 2, 3 and 10, are found [1]. Exon 10 encodes a microtubule binding repeat in the C-terminus in addition to three microtubule binding repeats existing in all tau isoforms. Exons 2 and 3 encode N-terminal insertions. Over 30 potential phosphorylation sites are identified in the tau molecule. Most of these phosphorylation sites are located in the flanking regions around the microtubule binding repeats and affect microtubule binding [2], [3].

Tau pathology is a defining feature in several neuropathological disorders called tauopathies [4]. They are characterized by tau aggregation into abnormal, beta-sheet containing filaments (PHFs) in neural cells and accompanied by a loss of axonal subcellular tau compartmentalization, by tau hyperphosphorylation and by changes in other posttranslational modifications. Whereas most tauopathies such as Pick's disease, corticobasal degeneration, progressive supranuclear palsy and Alzheimer's disease (AD) are not linked to mutations, frontotemporal dementia with parkinsonism linked to chromosome 17 is caused by autosomal dominant mutations in the MAPT gene [4].

Tau protein in its natively unfolded form is hydrophilic, highly soluble and degraded in an ubiquitin independent manner by the core (20S) of proteasome [5], [6]. The molecular incident responsible for triggering tau aggregation in sporadic tauopathies is unknown. The tau protein amino acid (aa) sequences most prone to convert into beta-sheet have been identified in the PHF6 and PHF6* sequence [7]. It is assumed that alterations in charge distribution in these amino acids may play a role in transition to a secondary structure.

Recent studies implicate that in addition to the tau protein expression level and turnover aberrant posttranslational modifications of tau protein such as phosphorylation [8], O-GlcNAc glycosylation [9] and oxidation [10] are involved in initiating tau aggregation. Protein–protein interaction that can induce secondary structure elements in natively unfolded proteins may also be a candidate for tau beta-sheet formation and tau aggregation. Further, deficiencies in the cytoplasmic chaperone system such as heat-shock protein function of hsp70/90 [11] and peptidyl-prolyl-isomerase activity of Pin1 have been implicated in tau pathology [12], [13].

For a better understanding of the molecular mechanisms behind tau pathology we screened for tau protein interaction partners, which may affect tau conformation or posttranslational modification. Using a cytoplasmic interaction screen we identified axotrophin also named MARCH7 as tau-interacting protein. Axotrophin belongs to the family of membrane-associated RING-CH (MARCH) proteins, although no transmembrane domains for axotrophin are recognizable [14].

Human axotrophin comprises a C_4_HC_3_-RING-variant domain in the C-terminal region similar to zinc binding RING domains of some ubiquitin ligases, also called E3 ligases [15]. Axotrophin is enriched in stem cells, neuronal cells and spermatids [14], [16], [17]. Axotrophin null mice are viable and fertile but show premature neuronal degeneration and defective development of the corpus callosum [18]. Axotrophin is functionally linked to the proteins Foxp3 and leukemia inhibitory factor. Thereby, axotrophin is believed to have an influence on T cell replication and immunotolerance with increased axotrophin expression in immunotolerance [19], [20].

Ubiquitination requires the concerted activity of E1, E2 and E3 enzymes. Ubiquitin is activated by ubiquitin activating enzyme (E1), and then transferred to an ubiquitin conjugating enzyme (E2) via thioester linkage. E3 ubiquitin ligases are responsible for specificity in substrate recognition either functioning solely as a scaffolding protein or additionally as a carrier of the activated ubiquitin. Ubiquitination of substrates can initiate a variety of downstream processes.

Poly-ubiquitination with a K48 linkage is the canonical pathway for proteasomal degradation of cytoplasmic proteins. Mono- or di-ubiquitination can induce internalization and trafficking, transcription and signal transduction depending on the ubiquitin linkage and length of the ubiquitin chain [21], [22].

For neurodegenerative diseases like AD there is evidence that the ubiquitin-mediated proteasomal degradation of proteins is disturbed: 1) inclusions of aggregated and ubiquitinated protein are found [23]; 2) proteasome activity in AD brains is lower than in age-matched controls [24], [25] and 3) mutations in enzymes of the ubiquitination cascade and ubiquitin are known to appear in neurodegenerative diseases [26], [27], [28].

The aims of this study are (i) the characterization of axotrophin regarding its subcellular localization and (ii) its potential E3 activity and (iii) the investigation of the tau axotrophin interaction with tau as a presumable substrate of axotrophin.

## 2 Materials and methods

### 2.1 Plasmids

The pGex-6P1 plasmid was purchased from Amersham Pharmacia Biotech. The pEGFPC2 and the pDSRed2 plasmid were purchased from Clontech. For PCR amplification of axotrophin and hook3 human fetal brain marathon-ready cDNA (Clontech) has been used. Other cDNAs were amplified from image clones. The integrity of all constructs was confirmed by automated sequencing. The prk172 tau plasmids are courtesy of M. Goedert, Cambridge, UK. The pGex-Hdm2 plasmid is courtesy of M. Scheffner, Konstanz, Germany [29].

### 2.2 Yeast two-hybrid screen

CytoTrap two-hybrid system (Stratagene) was used to identify new tau interacting proteins. Screening was performed according to the manufacturer's protocol. Full-length human tau protein 2N4R isoform cDNA (441aa) was cloned into bait vector pSos for expression of hSos–tau fusion-protein. CytoTrap human fetal brain cDNA library (Stratagene) cloned into target vector pMyr was used for screening. Positive clones were sequenced. For verification and further characterization of axotrophin interactions the DupLEX yeast two-hybrid assay was performed according to the manufacturer's recommendations (OriGene). Supplemental Table I summarizes properties of the used constructs. As negative control for interaction pJG-neg plasmid was used and for positive interaction control plasmids pBait and pTarget from the DupLEX system were used.

### 2.3 Co-immunoprecipitation

Vectors pEG-tau and pJG-axo were introduced into yeast strain EGY48 (*MATα trp1 his3 ura3 leu2:6 LexAop-LEU2*). After protein expression was induced by galactose cells were harvested and protein extracted in lysis buffer (50 mM Tris–HCl, pH 7.5, 10 mM EDTA, 1 mM EGTA, 5% (v/v) glycerol, 5 μg/mL leupeptin, 5 μg/mL pepstatin, 1 mM PMSF, and complete protease inhibitor cocktail by Roche). After pre-clearing with Protein-G Dynabeads (Invitrogen) in immunoprecipitation buffer (50 mM Tris–HCl, pH 7.5, 300 mM NaCl, 10 mM EDTA, 1 mM EGTA, 5% (v/v) glycerol, 1% (v/v) Triton X-100, 5 μg/mL leupeptin, 5 μg/mL pepstatin, 1 mM PMSF, and complete) LexA–Tau fusion protein was precipitated with Protein G-coupled mouse anti-LexA Dynabeads. Beads were washed and proteins were eluted by adding SDS gel loading buffer and 5 min incubation at 95 °C. Proteins were resolved by SDS-PAGE followed by Western Blot analysis using rabbit anti-tau antibody BR134 (1:1000) (1) and rabbit anti-AxoCT antibody, goat anti-rabbit alkaline phosphatase conjugate (Chemicon) and chemiluminescence detection. Anti-AxoCT is a polyclonal affinity-purified rabbit antibody directed against the C-terminus of axotrophin. The antibody was raised in a rabbit by immunization with KLH-conjugated peptide ARTLQAHMEDLETSED and isolated by an affinity column harboring the immobilized peptide.

### 2.4 Expression and purification of recombinant proteins

Overnight colonies of *Escherichia coli* BL21-CodonPlus (DE3)-RIL transformed with pGex-AxoRing (aa 549–704), pGex-AxoRingMut (aa 549–704, C552S, C555S), pGex-KLC1, pGex-PP2Ba, pGex-Hdm2 and tau plasmids prk172-ht40 (441aa, 2N4R isoform), prk172-ht46 (383aa, 0N4R isoform) or prk172-ht37 (410aa, 2N3R isoform) were inoculated into 1 L 2xTY containing 100 μg/mL ampicillin and 30 μg/mL chloramphenicol and grown at 37 °C. When OD_600_ reached 1.0, protein expression was induced with 0.5 mM IPTG for 2 to 4 h at 37 °C. The cells were harvested by centrifugation at 4500 ×*g* for 10 min at 4 °C. Pellets of cells transformed with a pGex plasmids were resuspended in 1/40 volume of PBS with 1 μg/mL leupeptin, 1 mM PMSF and 100 μM DTT. Pellets of cells transformed with a prk172 construct, expressing an isoform of human tau, were resuspended in 1/40 volume of lysis buffer (50 mM Tris–HCl, pH 7.4, 5 mM EDTA, 1 mM PMSF, 0.1 mM DTT). Resuspended pellets were lysed via sonication. To improve solubilization of protein 1% (v/v) Triton X-100, lysozyme (4000 U/mL) and benzonase (4 U/mL) were added for 1 h, slowly shaking at 4 °C. Lysate was cleared by centrifugation at 30,000 ×*g* for 20 min at 4 °C. GST fusion proteins KLC1, PP2Ba and Hdm2 were purified by adding glutathione-sepharose 4B to lysate supernatant for 2 h, slowly shaking at 4 °C. After washing sepharose beads several times with PBS, bound protein was cleaved from GST-tag by adding cleavage buffer (50 mM Tris–HCl, pH 8.0, 150 mM NaCl, 1 mM EDTA, 1 mM DTT) containing 10 U prescission protease per mL (GE Healthcare). Eluted proteins were dialyzed against dialysis buffer (5 mM MOPS, pH 7.0, 50 mM NaCl, 0.1 mM EDTA and 0.1 mM PMSF) for 4 h at 4 °C. All expressed GST axotrophin fragments led to formation of inclusion bodies and were found in the pellet after clearing the lysate by centrifugation. For purification of GST axotrophin fragments pellet was washed four times with washing buffer (2 M urea, 2% (v/v) Triton X-100). Thereafter pellet was extracted in urea buffer (7 M urea, 50 mM Tris–HCl, pH 7.0, 0.2 M NaCl, 50 μM ZnO(Ac)_2_, 5 mM DTT, 0.5 mM PMSF, 5% (v/v) glycerol) for 2 h and centrifuged at 30,000 ×*g* for 30 min at room temperature. The remaining pellet after urea wash was solubilized with guanidine buffer (urea buffer with 6 M guanidine hydrochloride instead of urea) for 2 h and cleared by centrifugation at 30,000 ×*g* for 30 min at room temperature. Guanidine hydrochloride supernatant was dialyzed against urea buffer for 4 h and pooled with urea supernatant followed by a slow sequential buffer exchange to refolding buffer (50 mM Tris–HCl, pH 7.5, 50 μM ZnO(Ac)_2_, 5 mM DTT). To remove precipitates, protein solution was centrifuged at 40,000 ×*g* for 20 min at 4 °C. For tau protein purification a modified procedure of Yoshida and Goedert was used [30]. Briefly, lysis supernatant of *E. coli* expressing an isoform of human tau was applied to a diethylaminoethyl cellulose column (DE52, Whatman) two times. Flow through was applied to a phosphocellulose column (P11, Whatman). Phosphocellulose was washed with lysis buffer and tau protein was eluted from the column in 5 mL fractions of 0.1 M, 0.2 M, 0.3 M, 0.4 M and 6 × 0.5 M NaCl. Fractions were analyzed via SDS-PAGE. Cleanest fractions were pooled, adjusted to a 50% saturation of ammonium sulfate and mixed at 4 °C for 20 min. Protein precipitate was harvested by centrifugation at 30,000 ×*g* for 30 min at 4 °C and resuspended in dialysis buffer (5 mM MOPS, pH 7.0, 50 mM NaCl, 0.1 mM EDTA and 0.1 mM PMSF). To resuspended protein 100 mM NaCl and 1% (v/v) β-mercaptoethanol were added, solution was heated to 100 °C for 10 min and centrifuged at 40,000 ×*g* for 10 min at 4 °C. Tau protein containing supernatant was dialyzed against dialysis buffer for 4 h at 4 °C.

### 2.5 In vitro ubiquitination assay

Enzymes and chemicals for ubiquitination were obtained from Biomol. Ubiquitination was performed according to the manufacturer's recommendations, with some modifications: scaling down the total reaction volume from 50 μL to 12.5 μL, using twofold volume of E3 enzyme (axotrophin) and target protein (tau, PP2Ba, KLC1) and extension of incubation time from 30–60 min to 3–4 h. Auto-ubiquitination of axotrophin was performed with each of the provided E2 enzymes and biotinylated ubiquitin without adding a target protein. Afterwards ubiquitination mixture was quenched by adding 12.5 μL 2 × non-reducing gel loading buffer and heated for 5 min to 80 °C. Tau protein ubiquitination was performed using UbcH5c as E2 enzyme, Axotrophin RING-variant domain as E3 enzyme, ubiquitin or methylated ubiquitin and biotinylated tau protein. Tau protein was biotinylated on its cysteine residue using maleimide-PEG2-biotin (Pierce). After ubiquitination tau protein was captured with magnetic streptavidin-beads and heated in 2 × non-reducing gel loading buffer for 5 min to 80 °C. The samples were separated via SDS-PAGE, blotted to PVDF membrane and analyzed by immunolabeling with peroxidase-conjugated extravidin (Sigma-Aldrich), polyclonal rabbit anti-human tau (Dako), polyclonal rabbit anti-ubiquitin (Dako) and donkey anti-rabbit IgG linked to peroxidase (GE Healthcare).

### 2.6 Binding of ubiquitinated tau to microtubules

For microtubule assembly cytoplasmic supernatant of tau knock-out brain [55] was prepared in assembly puffer (0.1 M MES pH 6.4, 2 mM EGTA, 0.5 mM MgCl_2_, 0.1 mM EDTA, complete protease inhibitor cocktail, 10 μM UCH-L1 inhibitor, 10 μM UCH-L3 inhibitor, Calbiochem) and supplemented with 20% glycerol and 1 mM GTP. After addition of 10 μL ubiquitinated 2N4R tau (ubiquitination performed for 4 h at 37 °C in the presence of the following enzymes: E1, UbcH5c, Axotrophin RING-variant domain) or 10 μL non-ubiquitinated 2N4R tau (ubiquitination mix without UbcH5c) microtubule assembly was incubated for 60 min at 37 °C. Microtubules were separated from supernatant by centrifugation (30,000 ×*g*, 30 min, 37 °C) through sucrose cushion and subsequently washed two times with prewarmed BRB80 buffer (80 mM PIPES pH 6.8, 1 mM MgCl_2_, 1 mM EGTA, 1 mM DTT, 1 mM GTP). Microtubule pellet was disassembled on ice in assembly buffer and boiled 5 min to remove tubulin by centrifugation. After adding 2 × gel loading buffer containing 6 M urea to assembly supernatant and heatstable microtubule pellet proteins were resolved via SDS-PAGE followed by Western Blot analysis using polyclonal rabbit anti-human tau (Dako) and donkey anti-rabbit IgG linked to peroxidase (GE Healthcare).

### 2.7 Cell culture and transfection

COS-7 and N2A cells were maintained in DMEM-Ham's F-12 supplemented with 10% fetal bovine serum (FBS), non essential amino acids (Biochrom AG) and 50 μg/mL gentamycin (PAA Laboratories) in a 5% CO_2_ environment at 37 °C. For transfection cells were plated in DMEM-Ham's F12 supplemented with 2% FBS and non essential amino acids over night. The following axotrophin cDNA fragments cloned into the Ecl136/SalI site of pEGFPC2: axoFL (aa 1–704), axoN1 (1–625), axoN2 (1–541), axoN3 (1–344), axoC1 (333–704), axoC2 (542–704) were used. The cells were transfected with pEGFP-axotrophin constructs with Lipofectamine 2000 (Invitrogen) according to manufacturer's recommendation. Primary neuronal cultures were prepared from E16 Wistar rat hippocampus using standard protocol [31] and cultured as described [32] except for seeding cells in neurobasal medium with B27 supplement (Invitrogen), 0.5 mM l-glutamine, 50 μg/mL gentamycin and 1% FBS for better attachment of cells. Half of the medium was renewed twice a week. After 10 days *in vitro* the cells were cotransfected with pRed-Tau and pEGFP-axotrophin using Lipofectamine 2000 (Invitrogen). Cultured and transfected cells were washed with PBS, fixed with 4% paraformaldehyde and 4% saccharose for 15 min at room temperature. Fixed cells were permeabilized with PBS and 0.2% (v/v) Triton X-100 for 10 min at room temperature. Nuclei of transfected COS-7, N2A and rat primary neuronal cells were stained with 4′,6-diamidino-2-phenylindole dihydrochloride (DAPI, Molecular Probes). Fluorescence localization was analyzed by laser-scanning confocal microscope (Zeiss, Heidelberg, Germany).

### 2.8 Immunohistochemistry

Human temporal cortex (Brodman area 22) and hippocampus of five control brains (age 73.6 ± 5.4 years) and five AD brains Braak stages II–VI (77.6 ± 5 years) were obtained at autopsy. The post-mortem diagnosis of AD is based on the recommendations of the National Institute on Aging (NIA)-Reagan Institute [33]. Human brain tissue was processed as described [13]. Briefly, endogenous peroxidase activity was quenched in 1% H_2_O_2_ and non-specific binding sites were masked by 1 h incubation in a blocking solution containing 2% BSA, 1% normal donkey serum and 0.01% Triton X-100 in TBS. Free-floating sections were incubated overnight (4 °C) with 1 μg/mL anti-AxoCT antibody. Immunoreactivity was visualized using the biotin–avidin system (biotinylated goat anti-rabbit IgG, 1:1000, Jackson ImmunoResearch Laboratories; biotinylated donkey anti-rabbit Ig, 1:1000; Amersham; streptavidin peroxidase VECTASTAIN ABC Kit, Vector Laboratories) and 3,3′-diaminobenzidine as chromogen. Tau knock-out mice (stock no. 004779) [55] and human tau gene transgenic mice, expressing all human isoforms (stock no. 004808) [56] were obtained from Jackson Laboratories (Bar Habor), and interbred. The mice were housed on an artificial 12 h light/dark cycle under conditions of constant temperature (22 °C) and humidity (50%). Animals received water and altromin standard diet ad libitum. Sibling animals at an age of 90 days of both genotypes were used for analysis. Mouse brain tissue was processed as described [34] and staining procedure for human tissue was applied. Immunoreactivity in cytoplasmic and nuclear compartments was semiquantitatively evaluated by measuring an optical density profile across each cell (20 cells each, on three slices) using ImageJ. The ratio of cytoplasmic versus nuclear immunoreactivity was calculated by averaging maximum grayscale in a 1 μm window in the cytoplasm and the nucleus.

### 2.9 Nuclear and cytosolic protein extraction from human temporal cortex

Frozen human temporal cortices (Brodman area 22, profile of cases see Suppl. Table II) were homogenized in 7.5 vol/g cytoplasmic extraction buffer (DPBS pH 7.4 supplemented with, 1 mM MgCl_2_, 1 mM EDTA, 2 mM EGTA, 25 mM NaF, 10 mM beta-glycerophosphate, 1 mm Na_3_VO_4_, 2 μg/mL leupeptin, 0.5 mM calpain inhibitor I and complete protease inhibitor cocktail, Roche) using an ULTRA-TURRAX and cooling on ice. Cytoplasmic supernatant was obtained from a 60 min 100,000 *g* centrifugation step at 4 °C and mixed with glycerol to yield 10% final concentration and stored at − 80 °C. The pellet was re-extracted with 2 vol. using a high-salt buffer (1.3 M LiCl, 30 mM LiCO_3_, 5 mM Tris/HCl pH 7.4, supplemented with the same components like the cytoplasmic extraction buffer) with ULTRA-TURRAX homogenization. The supernatant obtained from a 60 min 100,000 *g* centrifugation step at 4 °C was mixed with glycerol to yield 10% final concentration and stored at − 80 °C. 15 μg protein was separated on each lane of a 10% SDS-PAGE and transferred to a PVDF membrane. Immunolabeling was processed using anti-axotrophin monoclonal antibody BB-8 (Santa Cruz Biotechnology) and anti-actin monoclonal antibody C4 (Sigma). Band signal strength was quantified using densitometric software TINA 2.09g (Raytest).

## 3 Results

### 3.1 Identification of a tau interacting protein, axotrophin

**Fig. 1:**
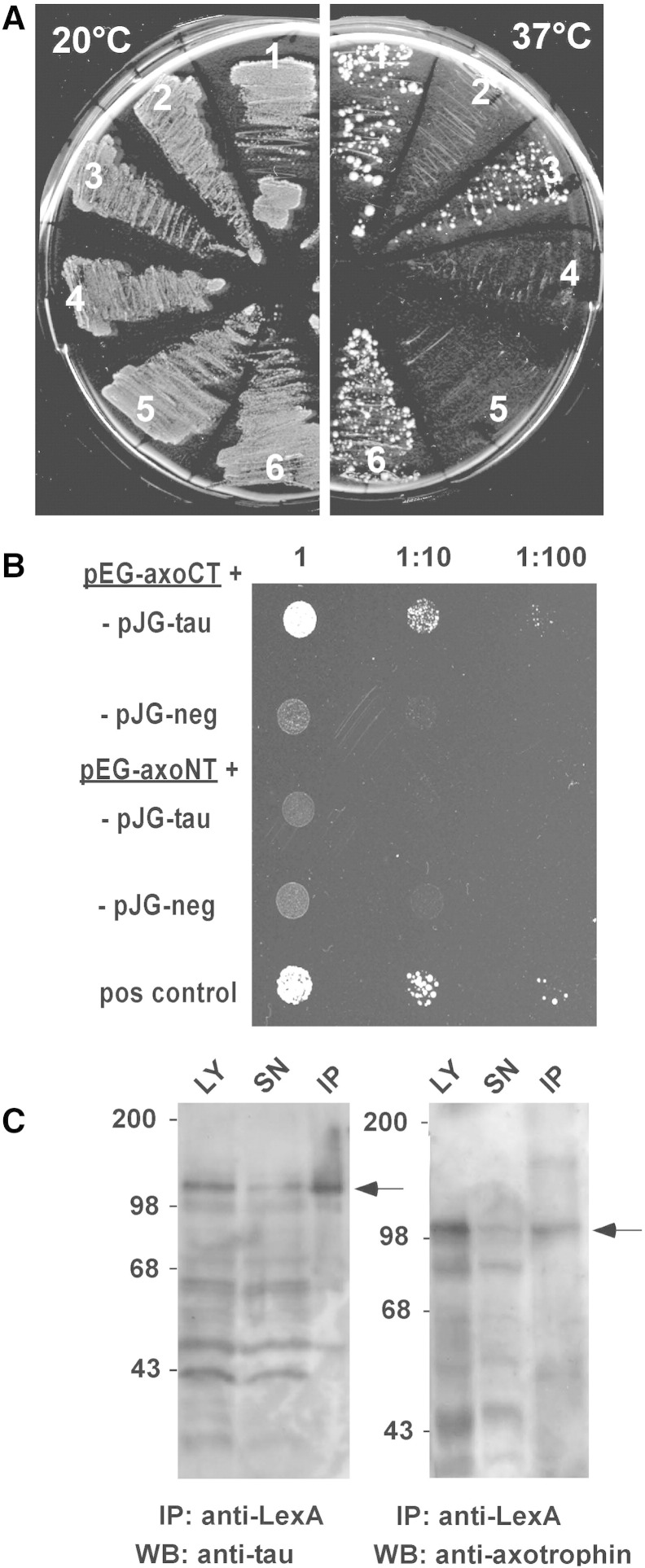
Tau interaction with axotrophin in yeast two-hybrid assays. (*A*) Identification of axotrophin as a tau-interaction partner employing the CytoTrap interaction assay. Transformed cdc25H cells grow under selective conditions (37 °C, galactose-Leu, -Ura), when an interaction between bait and prey is present (clones 1 and 3 pSOS-tau/pMyr-Axo, clone 6 pSOS-MAFB/pMyr-MAFB positive control). All clones grow at permissive temperature (25 °C) on galactose-Leu, -Ura. Clones 1, 3: pSOS-Tau + pMyr-Axo, clone 2: pSOS-Tau + pMyr-MAFB (negative control), clones 4, 5: pSOS-MAFB + pMyr-Axo (negative control), clone 6: pSOS-MAFB + pMyr-MAFB (positive control). (*B*) Confirmation and domain analysis of axotrophin–tau interaction using the DupLEX two-hybrid system. Two different axotrophin constructs were used as a bait to test for interaction with the prey tau protein. The axotrophin C-terminal part (pEG-axoCT) with the RING-variant domain comprising aa 552–682 shows a strong interaction with human tau (pJG-tau) indicated by growth on leucine-deficient medium. The long N-terminal part of axotrophin (pEG-axoNT) comprising aa 1–538 did not show an interaction with human tau. The prey protein hook3 was used as a negative control (pJG-neg). The positive control (pos control) was part of the DupLEX two-hybrid system displaying the interaction of the intracellular domain of the TGFβ-receptor and the FKBP18. (*C*) Co-immunoprecipitation of tau and axotrophin after coexpression of LexA–tau fusion protein and B42–axotrophin fusion protein in yeast cells. Tau protein from yeast lysate (Ly) was pulled down with matrix-coupled anti-LexA antibody and probed with tau antibody BR134 (left panel) in the immunoprecipitate (IP) and the supernatant after immunoprecipitation (SN). The precipitated tau fusion protein is marked with an arrow. Axotrophin immunoreactivity (right panel) was detected in the anti-tau immunoprecipitate by the polyclonal anti-AxoCT antibody (see arrow).

To identify tau interacting proteins, we employed the CytoTrap yeast two-hybrid assay. Unlike other yeast-based assays this test screens for interaction in the cytosolic compartment. We used the cDNA of the longest human tau isoform (2N4R isoform, 441aa) N-terminally fused to the SOS protein as a bait to screen a cDNA library constructed from human fetal brain (Fig. 1A). We obtained eleven positives, one of which was encoding aa 333–704 of *Homo sapiens* axotrophin/MARCH7 sequence (Gene ID 64844). Fig. 1A depicts reconstitution of the CytoTrap system with growth of yeast cells expressing SOS-tau protein and axotrophin at non-permissive temperature of 37 °C together with appropriate positive and negative controls. Sequence analysis displayed that axotrophin is a protein consisting of 704aa, comprising two main domains, a C-terminal RING-variant domain and a large N-terminal part with low sequence complexity but enriched in serine (observed frequency 20%, expected frequency 8.1% of full length axotrophin) and arginine (observed frequency 9.7%, expected frequency 4.2%). Full-length axotrophin can be found from fish to mammals (Suppl. Fig. 1). ClustalX sequence alignment reveals that the RING-variant domain is more conserved during evolution than the N-terminal serine/arginine-rich domain. In the N-terminal domain basic arginine-rich clusters such as aa 140–150, 276–300 and 391–411 have a higher degree of conservation than intervening sequences (Suppl. Fig. 1, numbering according human axotrophin sequence).

To validate the observed interaction between tau and axotrophin and to localize interacting domains we used the DupLEX yeast two-hybrid system as an alternative system, where protein–protein interaction has to occur in the nucleus. Therefore full-length axotrophin and N-terminal and C-terminal fragments of axotrophin were cloned into bait vector as well as cDNA of the longest human tau isoform into target vector and transformed into *Saccharomyces cerevisiae*. Since full-length axotrophin had autoactivating properties in the LexA-bait vector we tested different fragments and found that the fragments 1–538 comprising the N-terminal region (pEG-axoNT) and aa 552–682 comprising the RING-variant domain (pEG-axoCT) devoid of large acidic stretches were not autoactivating. Fig. 1B shows that tau protein is interacting with the 131aa RING-variant domain of axotrophin but not with the 538aa N-terminal region. This implicates that tau may be a substrate of the enzymatic activity of the RING-variant domain or a binding partner modulating interaction with E2 enzymes or other substrates.

Next, using full-length axotrophin we could show in yeast extract that immunoprecipitating LexA-tau co-precipitates axotrophin (Fig. 1C). Generally, detection of axotrophin in yeast lysates was complicated by the short half-life due to auto-ubiquitination (12). This can be seen by the loss of axotrophin signal in the lysate before (LY) and after precipitation (SN) (Fig. 1C right panel).

### 3.2 Axotrophin RING-variant domain acts as E3 ubiquitin ligase in collaboration with different E2 enzymes in vitro

**Fig. 2:**
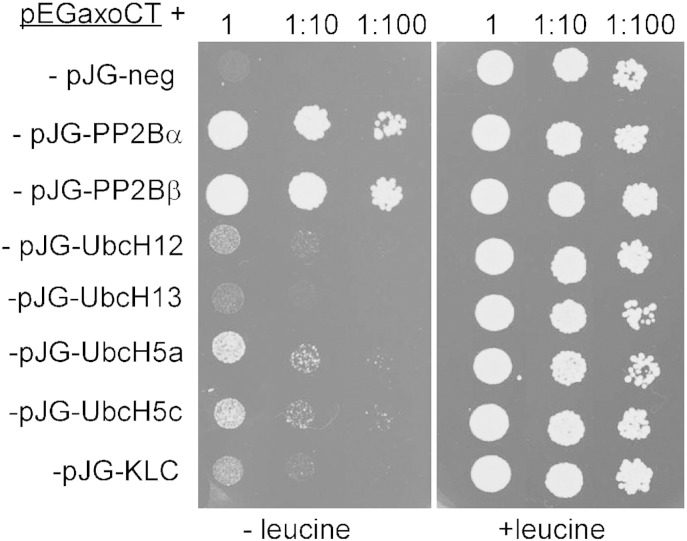
Screening analysis of proteins interacting with the axotrophin C-terminus comprising the RING-variant domain (aa 552–682). We analyzed E2 enzymes for interaction with axotrophin and search for other interaction partners by library screening and dual interaction testing using the DupLEX two-hybrid system. The yeast cells transfected with E2 enzymes UbcH5a and UbcH5c as activation-domain fusion proteins could grow on leucine deficient medium (left panel). This points to the UbcH5 family as E2 enzymes cooperating with the potential E3 ligase axotrophin. Weaker signals were obtained for the neddylation E2 enzyme Ubc12 and the ubiquitin chain elongating enzyme Ubc13. Two different isoforms of the protein phosphatase 2B (calcineurin) catalytic subunit (PP2Ba, PP2Bb) and kinesin light chain (KLC1) were identified as additional proteins interacting with the RING-variant domain or its flanking sequences.

**Fig. 3:**
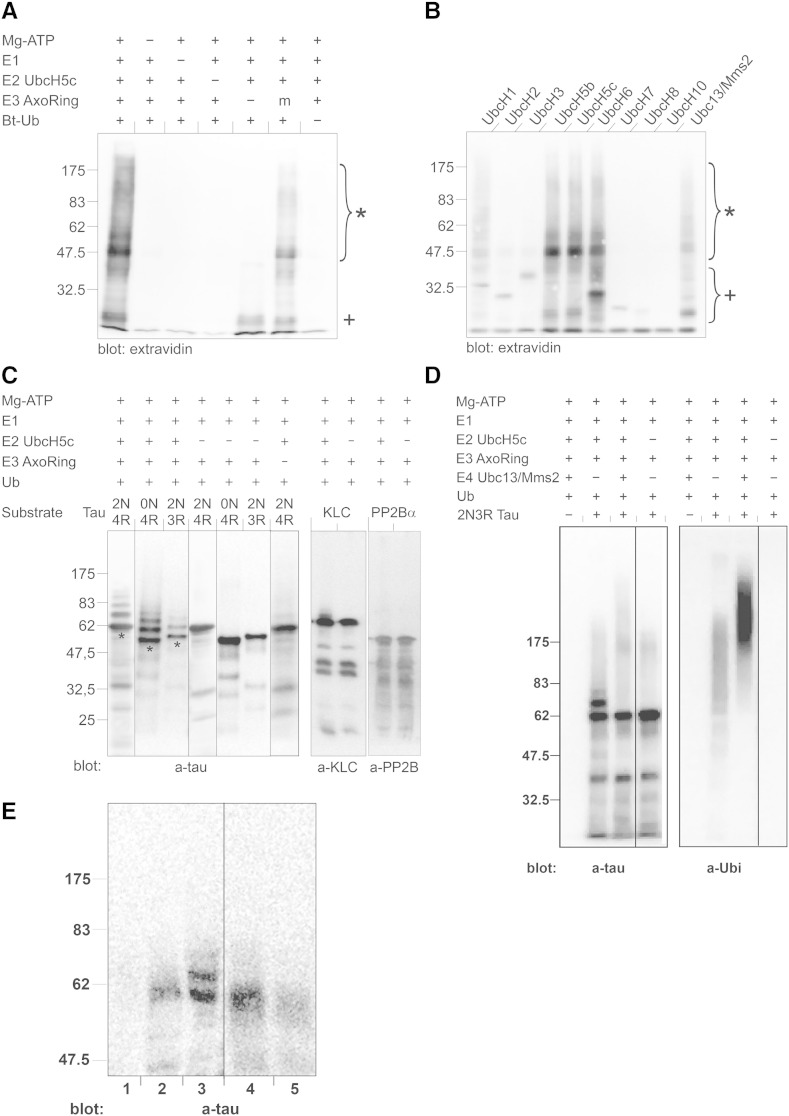
Reconstitution of axotrophin ubiquitin ligase activity after recombinant expression of the axotrophin C-terminus comprising the RING-variant domain in *E. coli*. (*A*) Testing of E3 auto-ubiquitinating activity of axotrophin RING-variant domain (AxoRing) by Western blotting. We used the UbcH5c E2 enzyme identified in the yeast two-hybrid assay to show that E3 auto-ubiquitinating activity of the RING-variant domain is dependent on all components of the ubiquitination cascade such as the presence of ATP, E1, E2 and ubiquitin. E3 auto-ubiquitinating activity of the axotrophin RING-variant domain is strongly diminished if the first two cysteines of the domain are substituted by serine residues (m, 552CRIC/552SRIS). The use of biotinylated ubiquitin (Bt-Ub) allowed the detection of ubiquitinated proteins by extravidin-HRP. (*B*) Screening for additional E2 enzymes capable to reconstitute axotrophin E3 ligase activity. In agreement with the yeast two-hybrid analysis the UbcH5 family was identified as the E2 enzymes conferring the highest enzymatic activity to axotrophin. In addition axotrophin E3 ligase activity was also dependent on UbcH6 and to a lesser extent on Ubc13, already identified by yeast two-hybrid analysis. UbcH1 had still has residual activity. *: auto-ubiquitinated axotrophin, +: ubiquitinated E2 enzymes. (*C*) Axotrophin RING-variant domain ubiquitinates tau *in vitro*. Axotrophin RING-variant domain (AxoRing) was incubated with Mg-ATP, E1, E2 UbcH5c, ubiquitin and biotinylated human tau isoforms 2N4R, 0N4R, 2N3R or two other axotrophin-interacting proteins KLC1 or PP2Ba. After separating proteins via SDS-PAGE, immunoblotting using an anti-tau, anti-KLC1 or anti-PP2B was done. Lane 1 shows that ubiquitination of the longest human tau isoform 2N4R (62 kDa) produced four additional immunoreactive bands each shifted 7–8 kDa apart in addition to the unmodified protein (asterisk). In the control reactions lacking E2 enzyme (lanes 4–6) and E3 enzyme (lane 7) tau protein isoforms run unmodified as a single band. Lane 2 shows ubiquitination of 0N4R tau isoform, inducing four additional immunoreactive bands. The unmodified 0N4R isoform in lane 5 runs at 55 kDa. Ubiquitination of the human 2N3R tau isoform (that includes the N-terminal inserts but only three instead of four C-terminal microtubule binding repeats) in lane 3 produces only two additional tau bands. The unmodified 2N3R isoform in lane 6 runs at 58 kDa. Complete ubiquitination cascade with E2 UbcH5c leads to additional tau bands above the unmodified tau band, which are shifted each by about 7–8 kDa and represent ubiquitinated tau (lanes 1–3). Employing KLC1 or PP2Ba in the ubiquitination assay as substrates does not result in ubiquitin incorporation in these axotrophin-interacting proteins (compare lanes 8 and 9 and lanes 10 and 11). Ub: ubiquitin, molecular weight is indicated left in kDa. (*D*) Ubiquitin chain elongation of mono-ubiquitinated tau protein by E4-ligase Ubc13/Mms2. Depletion of shifted 2N3R tau bands in the presence of Ubc13/Mms2 (compare lanes 2 and 3) and increase in high-molecular weight tau smear, which is more visible with anti-ubiquitin antibody (compare lanes 6 and 7), which preferentially labels poly-ubiquitinated tau protein. There is some polyubiquitinated tau protein in the absence of Ubc13/Mms2 (lane 6). (*E*) Microtubule-binding assay of ubiquitinated 2N4R-tau. 2N4R-tau was ubiquitinated in the presence of Mg-ATP, E1, E2: UbcH5c, E3: axotrophin RING-variant domain and ubiquitin (lanes 2 and 3) or left unmodified by omission of UbcH5 in the reaction mix (lanes 4 and 5). Microtubule assembly in the presence of ubiquitinated 2N4R-tau or non-ubiquitinated 2N4R tau was performed and microtubules were pelleted. Lane 1 shows the absence of tau immunoreactivity in the microtubule pellet, when no exogenous tau is added. Tau species in supernatant (lanes 3 and 5) and microtubule pellet (lanes 2 and 4) were detected by anti-tau immunoblotting. Ubiquitinated tau, visible by shifted bands was detected mostly in the supernatant and not in the microtubule pellet.

The axotrophin C_4_HC_3_-RING-variant domain occurring in E3 ubiquitin ligases suggests that axotrophin itself may act as E3 ubiquitin ligase. Therefore, we investigated the potential E3 ubiquitin ligase activity of axotrophin and its association with relevant E2 enzymes. Due to the inherent instability of axotrophin in living cells we expressed and purified recombinant axotrophin C-terminus (aa 548–704) comprising the RING-variant domain from *E. coli* inclusion bodies and recovered enzymatic activity by a slow protein refolding process. For reconstitution of the ubiquitin ligase cascade we had to determine which E2 enzyme is the appropriate interaction partner for axotrophin. We performed a yeast two-hybrid assay to test interaction with E2 enzymes known to interact with RING-variant domains of other MARCH proteins. Here we identified members of UbcH5-family, UbCH12 and UbcH13 as interaction partners (Fig. 2). Therefore, we used UbcH5c as E2 enzyme together with other components of ubiquitination cascade (Mg-ATP, E1 ligase and biotinylated-ubiquitin) and with recombinant axotrophin RING-variant domain to assay for E3 ligase activity. Auto-ubiquitination of axotrophin detected by extravidin-HRP was used as readout of ligase activity. We verified specificity of the reaction by omission of each component and mutation of the axotrophin RING-variant domain (Fig. 3A). The linkage of ubiquitin to wild-type axotrophin RING-variant domain does not occur if one component of the ubiquitination cascade is absent. Replacement of the two cysteines 552/555 of the CRIC motif of axotrophin with serine residues should hamper binding of one of the catalytically essential zinc ions and reduce ligase activity. Although the coordination of the second zinc is undisturbed the mutated RING-variant domain has a strongly diminished ligase activity (Fig. 3A, compare lane 1 and lane 6). These data reveal that axotrophin RING-variant domain acts as an E3 ubiquitin ligase with the feature of auto-ubiquitination.

Having established an *in vitro* assay of ligase activity we performed a more thorough screening for potential E2 enzymes cooperating with axotrophin. Fig. 3B shows that beside UbcH5b/c, UbcH6 and to a lesser extent UbcH13 are supporting axotrophin E3 ligase activity because we observed a linkage of ubiquitin to axotrophin. UbcH1 (E2-25k) showed only weak activity and all other tested E2 enzymes (UbcH2, 3, 7, 8, 10) were inactive.

### 3.3 E3 ubiquitin ligase activity of axotrophin RING-variant domain leads to ubiquitination of tau in vitro

The observation that tau and axotrophin RING-variant domain interact led us ask, whether the E3 ubiquitin ligase activity of the axotrophin RING-variant domain can induce ubiquitination of tau. To answer this question we carried out an *in vitro* ubiquitination assay containing Mg-ATP, E1, E2 UbcH5c, ubiquitin, axotrophin RING-variant domain as E3 and human tau isoforms 2N4R, 0N4R, 2N3R as putative E3 ligase substrates. 2N4R is the longest human tau isoform including two N-terminal inserts and four microtubule binding domains (441aa). 0N4R contains four microtubule binding domains but the N-terminal inserts are absent (383aa). 2N3R includes two N-terminal inserts and three instead of four microtubule binding domains (410aa). We used these three human tau isoforms to ascertain if there are differences in exon-specific tau ubiquitination by axotrophin RING-variant domain.

When E2 enzyme UbcH5c is not added only the unmodified tau band is apparent: for 2N4R tau at 62 kDa, for 0N4R tau at 55 kDa and for 2N3R tau at 58 kDa (Fig. 3C, lanes 4, 5 and 6). The same occurs when E3 enzyme axotrophin is omitted (Fig. 3C, lane 7). With a complete ubiquitination cascade we see additional tau bands above the unmodified tau band, which are shifted each by about 7–8 kDa. For 2N4R and 0N4R tau up to four such shifted bands above the unmodified tau band are visible (Fig. 3C, lanes 1 and 2). The 2N3R isoform exhibits two shifted bands above the unmodified tau band (Fig. 3C, lane 3). These shifted tau bands and some higher molecular weight tau immunoreactivity are the result of tau ubiquitination by the axotrophin RING-variant domain. In a second ubiquitination assay we used methylated ubiquitin, deficient for ubiquitin chain elongation, and observed a similar banding pattern compared to unmethylated ubiquitin (Suppl. Fig. 2, compare lane 1 with lane 3 and lane 2 with lane 4). This result led us conclude, that the tau isoforms are preferentially mono-ubiquitinated at multiple sites by axotrophin affecting at least four lysine residues in four-repeat tau isoforms (0N4R, 2N4R) and two lysine residues in three-repeat 2N3R isoform. The additional ubiquitination sites of 0N4R and 2N4R tau in comparison to 2N3R tau allows us to assume, that these ubiquitination sites are localized in the second microtubule binding domain encoded by exon 10, which contains 5 lysine residues. The ubiquitin-linkage to tau protein is confirmed by Western blotting with anti-ubiquitin antibody (Fig. 3D). Furthermore, the addition of an E4 poly-ubiquitin ligase activity using Ubc13/Mms2 to the ubiquitination cascade leads to a depletion of the shifted tau bands and to appearance of high molecular tau species (Fig. 3D), strongly labeled by the anti-ubiquitin antibody, which preferentially binds to poly-ubiquitinated proteins.

Control ubiquitination of biotinylated 2N4R tau by recombinant E3 ligase Hdm2 in the presence of the complete ubiquitination cascade including UbcH5c did not induce tau ubiquitination (data not shown).

In attempt to find more potential axotrophin interaction partners or substrates, we used the LexA-axotrophin RING-variant domain fusion protein (pEG-axoCT) to screen an AD-related cDNA library and we identified the catalytical subunit of protein phosphatase 2B (PP2Ba, calcineurin) and kinesin light chain (KLC1) as positive clones in the yeast two-hybrid assay (Fig. 2). We purified both proteins after recombinant expression in *E. coli*. Unlike tau protein, no incorporation of ubiquitin occurred in these proteins in the presence of the complete ubiquitination cascade (Fig. 3C lanes 8, 9 and lanes 10, 11).

### 3.4 Multiple mono-ubiquitination of tau by axotrophin leads to a decreased microtubule binding capacity of tau

The multiple mono-ubiquitination of tau by the RING-variant domain of axotrophin suggests that the microtubule binding affinity of tau is affected by tau ubiquitination. To verify this assumption we assembled microtubules in the presence of ubiquitinated or non-ubiquitinated 2N4R tau protein obtained by incubation of tau in the presence of E1, axotrophin RING-variant domain (E3) and with or without UbcH5c (E2). Afterwards, the assembly supernatant containing unbound tau and the pellet containing microtubule-bound tau were separately analyzed via Western Blot (Fig. 3E). Mono-ubiquitination of 2N4R tau by the axotrophin RING-variant domain leads to additional tau bands above the unmodified tau band at 62 kDa. Ubiquitinated tau protein is found almost exclusively microtubule-unbound in the supernatant (Fig. 3E, lane 3). Non-ubiquitinated tau obtained by ubiquitination mix devoid of UbcH5c preferentially accumulates in the microtubule pellet (Fig. 3D, lane 4). This result shows that ubiquitin-modification of tau by the axotrophin RING-variant domain leads to a decreased microtubule binding affinity of tau.

### 3.5 Localization of tau and axotrophin in primary neurons, AD brain and tau knock-out mice

**Fig. 4:**
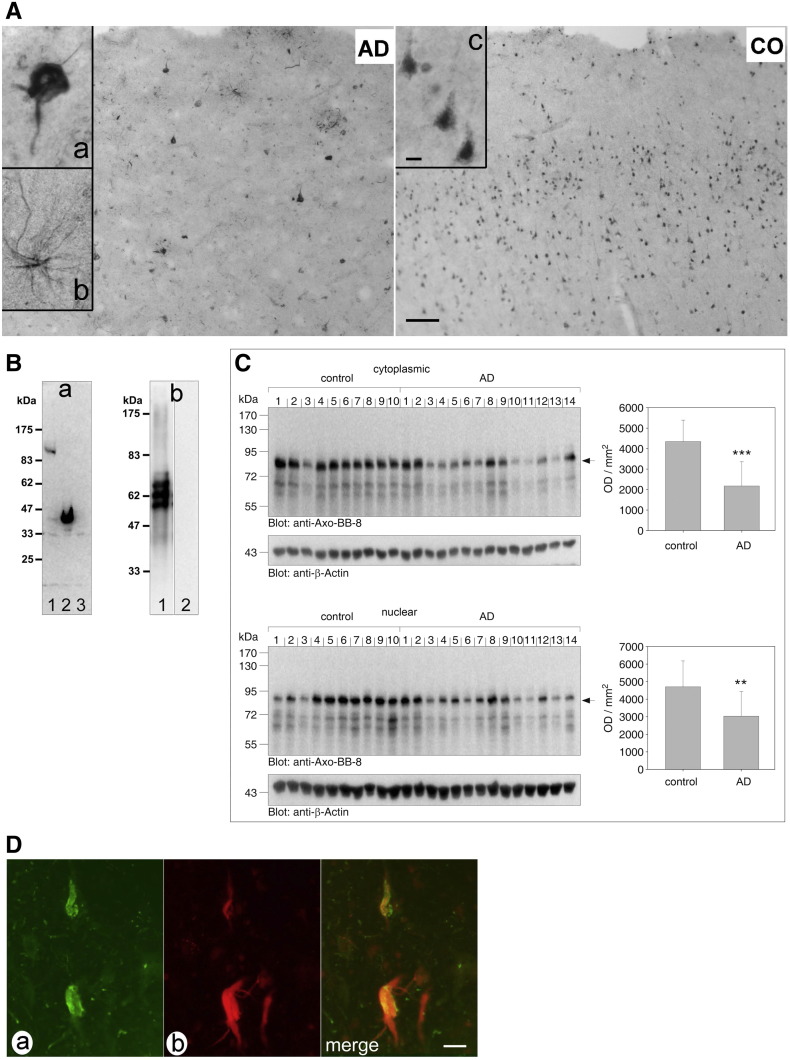
Axotrophin immunohistochemistry of human brain tissue. (*A*) Brain sections (area 22) were labeled with the affinity-purified anti-AxoCT antibody and visualized with DAB/Nickel. In control brain (CO, lower panel, scale bar: 100 μm) axotrophin is enriched in the nucleus of pyramidal cells in layer III and layer V and to a lesser extent in the cytoplasm and proximal dendrites of pyramidal cells (inset c, scale bar: 10 μm). In AD brain (AD upper panel, Braak stage V) the preferential nuclear localization is lost and axotrophin immunoreactivity is associated with pathological tau aggregates in dystrophic neurites around neuritic plaques and neurofibrillary tangles (inset a). In addition hypertrophic astroglial cells are labeled in AD brain (inset b). (*B*) Specificity of affinity-purified anti-AxoCT antibody. Panel a: COS7 cell lysate transfected with pEGFP-axoFL (lane 1), pEGFP-axoC2 (lane 2) and pEGFP (lane 3). Western blot analysis with anti-AxoCT antibody labels a 100 kDa protein in lane 1 corresponding to the GFP-axotrophin (aa 1-704) fusion protein and a 45 kDa protein in lane 2 corresponding to the GFP-axotrophin (aa 542-704) fusion protein. Endogenous axotrophin was undetectable. Panel b: PHF-tau prepared from temporal cortex of AD brain by the sarcosyl procedure was probed with antibodies Tau5/HT7 (lane 1) and anti-AxoCT (lane 2). Three immunoreactive bands corresponding to A68 PHF-tau were strongly labeled by the tau antibodies. The anti-AxoCT antibody does not cross-react with PHF-tau (lane 2). (*C*) Quantification of axotrophin in cytoplasmic and nuclear protein fractions obtained by differential protein extraction from temporal cortex (Brodmann area 22). Equal amount of protein was resolved by SDS-PAGE and labeled with a monoclonal axotrophin antibody. Signal intensity of the 80 kDa protein band was quantified by densitometry and revealed a significant loss of cytoplasmic and nuclear axotrophin protein in AD cases (Student's t-test, **p < 0.01, ***p < 0.001). (*D*) Co-localization of axotrophin and neurofibrillary tangles in the hippocampus of AD brain. Axotrophin was labeled by the polyclonal anti-AxoCT antibody followed by Cy3-linked anti-rabbit secondary antibody (panel b) and for the detection of tau aggregates monoclonal tau antibody AT8 (1:500) was used followed by Cy2-linked anti-mouse secondary antibody (panel a). Fluorescence labeling was analyzed by confocal laser scanning microscopy. Axotrophin is colocalized with tau aggregates in hippocampal neurons (panel merge, scale bar: 20 μm).

Tau filaments, a defining feature of several neurodegenerative diseases, have been reported to be ubiquitinated [35]. To analyze a possible association of axotrophin with tau proteins we prepared an affinity-purified rabbit antibody directed against a C-terminal axotrophin epitope ARTLQAHMEDLETSED conserved in mammals. The specificity of this polyclonal anti-AxoCT antibody was verified by Western blotting of axotrophin-transfected cell culture and no cross-reaction with hyperphosphorylated PHF-tau has been observed (Fig. 4B). Next, we analyzed axotrophin localization in temporal cortex and hippocampus of five controls and AD brains. Immunohistochemistry of human control brain tissue revealed a predominant nuclear staining of pyramidal cells of layer III and layer V in the temporal cortex (Fig. 4A CO and inset c) and other brain areas. In 30% of pyramidal cells axotrophin immunoreactivity extends into the apical dendrite.

In AD brain tissue the cytoplasmic and nuclear staining of neurons are largely abolished and tau aggregates such as neurofibrillary tangles and dystrophic neurites of senile plaques are labeled (Fig. 4A AD inset a). In addition, hypertrophic astroglial cells are stained (Fig. 4A AD inset b). Differential extraction of cytoplasmic and nuclear fraction of axotrophin and quantification on Western blot showed a loss of immunoreactivity of 50% and 36.3%, respectively (Fig. 4C). The intracellular co-localization of tau aggregates and axotrophin has been confirmed by immunofluorescence double-labeling and confocal laser scanning microscopy (Fig. 4D).

To visualize an interaction of tau and axotrophin in living cells we transfected rat primary neurons with RFP-tau and EGFP-axotrophin. Confocal laser scanning microscopy shows co-localization of tau and axotrophin in different neuronal compartments such as cell soma and at branching points of neurites (Suppl. Fig. 3).

**Fig. 5:**
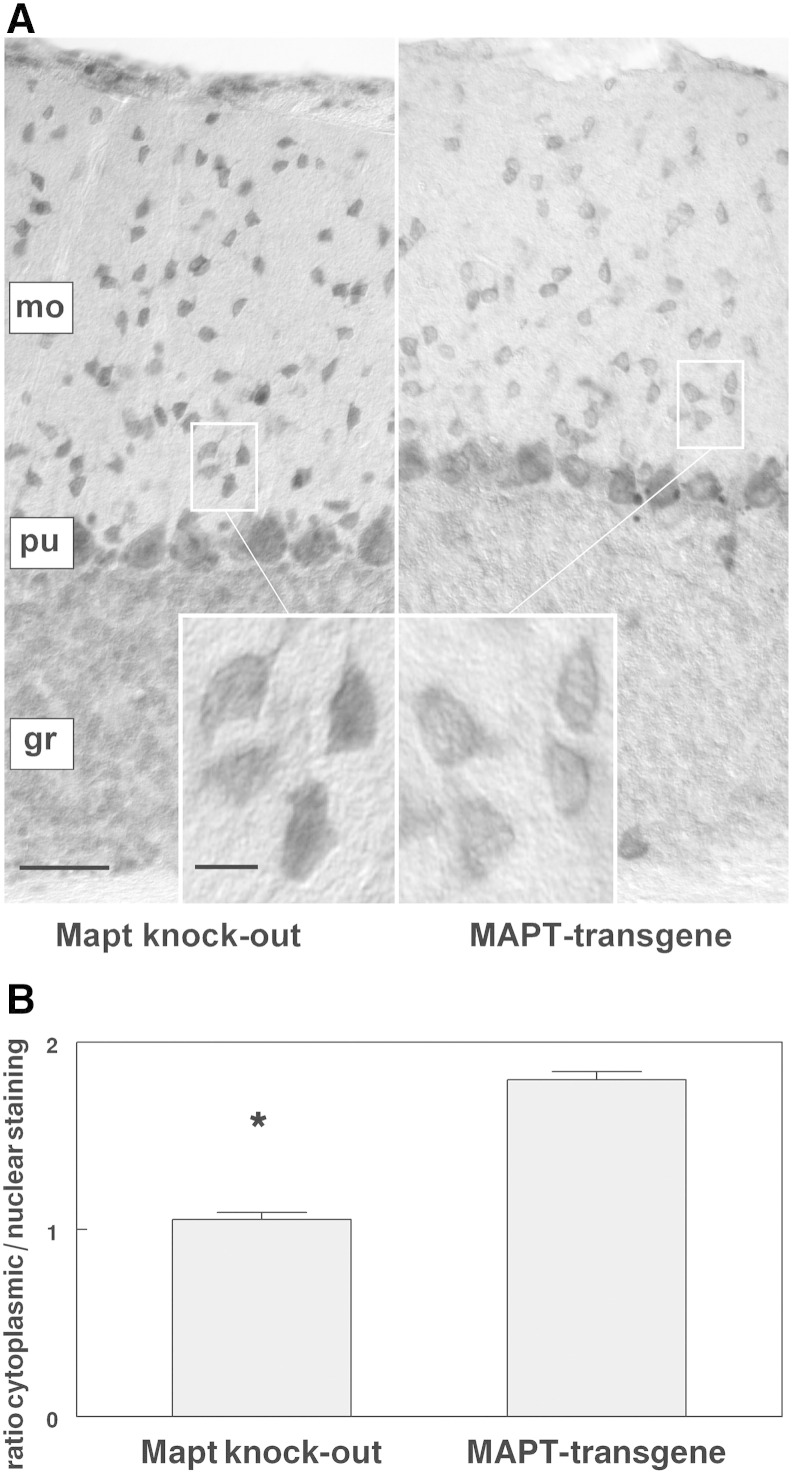
Axotrophin immunohistochemistry in cerebellum of tau knock-out and human tau gene transgenic mice. (*A*) Brain sections were labeled with the affinity-purified anti-AxoCT antibody and visualized with DAB/Nickel. Cerebellar axotrophin immunoreactivity is detected in neurons of the molecular layer (mo), Purkinje cell layer (pu) and granule cell layer (gr). Stellate neurons in the molecular layer and Purkinje cells are strongly labeled, whereas the granule cell layer exhibits a diffuse staining. Tau knock-out mice reveal a stronger nuclear axotrophin staining than mice expressing the human tau isoforms (human tau gene-transgenic mice, scale bar: 30 μm). The insets show that increased axotrophin staining in tau knock-out mice is due to a stronger axotrophin accumulation in the nucleus (scale bar 6 μm). (*B*) Semi-quantitative densitometric analysis of cytoplasmic and nuclear axotrophin immunoreactivity. There is a significant increase in nuclear axotrophin localization due to the absence of human tau expression in Mapt knock-out brains (Mann–Whitney U-test, p ≤ 0.05, n = 3).

Having shown the axotrophin co-localization with tau aggregates in AD tissue, we asked how the interaction partner axotrophin is affected if the other partner, tau protein, is missing. To this end we compared axotrophin immunohistochemistry in tau knock-out mice with human tau gene transgenic mice, expressing all human isoforms. Fig. 5A presents axotrophin immunoreactivity in the cerebellum, where axotrophin immunoreactivity is detected in neuronal cells. Stellate cells and purkinje cells are strongly labeled, whereas other cerebellar neurons such as golgi cells and granule cells are weakly stained. The absence of tau protein leads to a prominent nuclear localization of axotrophin in the tau knock-out mice. This contrasts with human tau gene transgenic mice, where the nuclear compartment of stellate and purkinje cells remains more spared of axotrophin immunoreactivity, for quantification see Fig. 5B. This leads us to the assumption that axotrophin may be sequestered by tau in the cytosolic compartment and this interaction diminishes axotrophin levels.

### 3.6 Subcellular localization of axotrophin

Tau protein is known to occur in the cytoplasmic and nuclear subcompartments and fulfills different functions in these locations [57] and detection of endogenous axotrophin in human and mouse brain tissue revealed also a dual cytoplasmic and nuclear localization (Fig. 4, Fig. 5). Having shown that tau protein is influencing the intracellular axotrophin location we asked, which subcellular targeting signals within the axotrophin amino acid sequence are present.

**Fig. 6:**
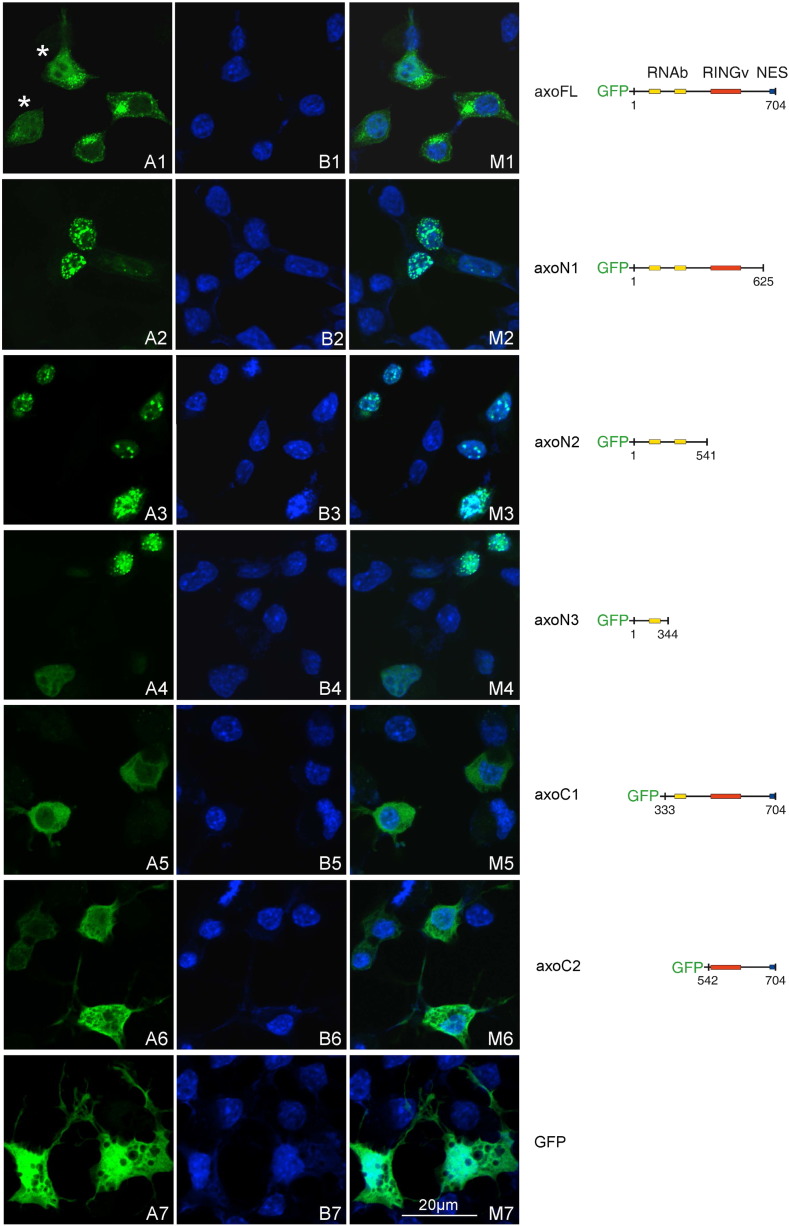
Subcellular localization of EGFP–axotrophin fusion proteins. N2A cells were transfected with constructs encoding different axotrophin domains and full length axotrophin as EGFP-fusion proteins. Confocal images of EGFP fluorescence (A1–A7), nuclear DAPI fluorescence (B1–B7) and merge EGFP- and DAPI-fluorescence (M1–M7) were obtained by laser scanning microscopy. Axotrophin expressed as full length protein has a cytoplasmic and nuclear localization (A1, B1). In the cytoplasm axotrophin resides in granular structures in close proximity to the Golgi apparatus. In addition picture A1 shows two cells (marked with asterisk) with more prominent nuclear localization. Upon deletion of aa 626–704 axotrophin exhibits a predominant nuclear localization in a nuclear speckle-like appearance (A2, B2). This nuclear speckle-like localization does change when further amino acids were removed from the C-terminal end such as deleting the RING-variant domain leading to a more heterochromatin associated localization (A3, B3), visible by the large overlap with DAPI stained nuclear foci. After deletion the complete C-terminal half yielding a 344aa construct comprising only the first of two putative DNA/RNA binding domains the nuclear axotrophin localization is confined to smaller nuclear subcompartments (A4, B4). In contrast deletion of aa 1–332, removing the first of two putative DNA/RNA binding domains (A5, B5) results in a predominant cytoplasmic localization without the granular appearance observed with full-length axotrophin (A1, B1). Further deletion of the second putative DNA/RNA binding domain resulting in a construct comprising the RING-variant domain and the C-terminus leads to a cytoplasmic localization of the EGFP-fusion protein with a reticular occurrence (A6, B6). For comparison the dual appearance of solely EGFP in the nuclear and cytoplasmic compartments is shown (A7, B7).

Transient overexpression of EGFP-tagged full-length axotrophin in primary neurons and cell lines showed a dual distribution by confocal laser scanning microscopy (Suppl. Fig. 3 and Fig. 6A1). To study which axotrophin sequences contribute to the different subcellular localizations, we transiently expressed different EGFP-tagged axotrophin domains: N-terminal domains (axoN1 aa 1–625, axoN2 1–541, axoN3 1–344) and C-terminal domains (axoC1 aa 333–704, axoC2 542–704) in N2A mouse neuroblastoma cells (Fig. 6) and in COS-7 cells (data not shown). Fig. 6A1 depicts punctuate cytoplasmic and diffuse nucleoplasmic distribution of full-length axotrophin in N2A cells. Deletion of the C-terminal 78aa stretch (axoN1) leads to a pronounced nuclear localization (Fig. 6A2). The same localization is found in axoN2, where additionally the RING-variant domain was removed (Fig. 6A3). AxoN1 localizes to bright, irregularly shaped structures resembling nuclear speckles. AxoN1 fragment contains 861aa and is too large to be targeted to the nuclear compartment without active nuclear import. This provokes the questions, where the nuclear localization signal (NLS) in axoN1 and the nuclear export sequence (NES) in the deleted 78aa fragment are localized. Using prediction algorithms for NLS no candidate sequence was found. NES prediction identified the sequence LNLEDFD right after the RING-variant domain, but this sequence is still included in axoN1. The skewed amino acid composition of the N-terminal region rich in serine and arginine resembles serine/arginine-rich (SR) proteins, which also travel between the nucleus and the cytoplasm. This led us test whether axotrophin has RNA recognition motifs. RNAbindR [36], [37] predicts two RNA-binding stretches within aa 76–109 and 387–476 in axotrophin. To verify the significance of the RNAbindR prediction, we expressed axotrophin aa 1–344 (axoN3). AxoN3 is targeted to punctuate nuclear structures too, arguing for a predominant role of the first RNA-binding motif (aa 76–109) for nuclear targeting (Fig. 6A4). In an opposite approach, we deleted aa 1–332 of axotrophin (axoC1) and found a diffuse preferential cytoplasmic localization (Fig. 6A5). This emphasizes the functional relevance of the first RNA-binding motif, of which loss is not compensated by the remaining RNA-binding motif. Expressing the C-terminus starting with the RING-variant domain (axoC2, aa 542–704) EGFP-fluorescence reveals a more reticulate cytoplasmic appearance and in addition can be found at the plasma membrane and cellular extensions (Fig. 6A6). Fig. 6A7 presents for comparison the cytoplasmic localization of EGFP protein which also gains access to the nucleus by diffusion due to its small molecular size.

## 4 Discussion

The aim of the present study was to find tau-interacting proteins, which can affect tau conformation or posttranslational modification.

We have identified and characterized axotrophin, a protein that binds and preferentially mono-ubiquitinates tau protein. The tau interaction domain of axotrophin is localized to a C-terminal 131aa fragment containing the RING-variant domain.

Loss of nuclear localization and accumulation of axotrophin in neurofibrillary tangle containing neurons of AD brains indicates a relevance of axotrophin-tau interaction in the pathogenesis of this disease.

Axotrophin is an atypical member of the family of the membrane-associated RING-CH (MARCH) proteins [15], due to its lack of a transmembrane domain and the C-terminal localization of the RING-variant domain. Viral E3 ligases such as K3 and K5 harboring a RING-variant domain have been shown to be involved in immune evasion by downregulating MHC class I protein [38].

Ubiquitin ligase activity of axotrophin has been demonstrated by Nathan et al. [14] by the high level of auto-ubiquitination, causing rapid axotrophin degradation. Decreasing axotrophin auto-ubiquitination by overexpression of ubiquitin hydrolases USP7 and USP9x increased the axotrophin level [14]. Because of the low endogenous levels of axotrophin we decided to characterize the enzymatic activity by making use of recombinant axotrophin. We succeeded with recombinant expression and purification of the enzymatically active axotrophin fragment. Testing different E2 enzymes for axotrophin interaction in a yeast two-hybrid assay we identified UbcH5c as a strong interactor, stimulating also highest axotrophin auto-ubiquitination.

Other E2 enzymes with weaker axotrophin interaction, including UbcH12 and UbcH13, UbcH6 and UbcH1 (Hip2, E2-25K), were less efficiently recruited by axotrophin in the ubiquitination assay resulting in low levels of auto-ubiquitination.

Preference of RING-variant domain of viral ubiquitin ligase K3 for UbcH5 and UbcH13 was elucidated by Dodd et al. [39], whereas two recent investigations described UbcH1 as also an important axotrophin activator [14], [40]. Whereas most membrane-bound MARCH proteins are involved in membrane-receptor downregulation such as MHCI and MHCII [41], the substrate of axotrophin/MARCH7 ligase activity remains elusive. Flierman et al. [40] suggest that MHCI may serve as a target of axotrophin. Recently, Gao et al. [42] found that axotrophin mRNA and gp190 subunit of leukemia inhibitory factor (LIF) receptor are inversely correlated, implying that gp190 can be ubiquitinated by axotrophin. We showed that endogenous and overexpressed full length axotrophin resides mainly in the nuclear and cytosolic compartments, where it also can interact with non-membrane bound substrates. We report that the microtubule-associated tau protein is such a cytoplasmic protein, which binds to axotrophin and is a substrate of axotrophin E3 ligase activity. We present several lines of evidence for this interaction, (i) positive interaction in two different yeast two-hybrid systems, (ii) association found by co-immunoprecipitation, (iii) axotrophin association with tau protein aggregates in Alzheimer's disease, (iv) altered axotrophin expression/localization in tau knock-out mice and (v) tau mono-ubiquitination by axotrophin *in vitro*.

Mono-ubiquitination of tau protein by axotrophin is unlikely to be a proteasome targeting signal but rather affects affinity of tau to microtubules. Roughly half of the 44 potentially ubiquitin accepting lysine residues of the 2N4R tau isoform are comprised within the four microtubule-binding repeats. More ubiquitin was incorporated into four-repeat tau than three-repeat tau, suggesting that at least one ubiquitin acceptor lysine resides in exon 10. Therefore we assume a tau-microtubule-affinity regulatory activity of axotrophin, which has a stronger effect on four-repeat than on three-repeat tau protein. We could show that addition of ubiquitin side-chains prevents tau from binding to pre-formed microtubules.

The preferential ubiquitination of four-repeat tau by the Hsc70-interacting protein CHIP was also noted by Hatakeyama et al. [43]. In contrast to axotrophin, CHIP induces poly-ubiquitination of tau regulating tau degradation [44], [45]. Poly-ubiquitination sites in PHF-tau from AD brain were identified as Lys254, Lys311 and Lys353 [35], [46], whereas sumoylation was confined to Lys340 [47]. Tau poly-ubiquitination is acknowledged to be a late process in the pathogenesis of Alzheimer's disease and in an animal model of tau pathology [48], [49]. Whereas multiple mono-ubiquitination of tau by axotrophin could be a physiological event regulating microtubule binding affinity of tau it may also provide the basis for ubiquitin chain elongation by so called E4-ligases.

One morphological feature of axotrophin knock-out animals is reduced axogenesis resulting in the absence of the corpus callosum [18]. Tau protein is enriched in the axonal compartment, where it functions as a regulator of microtubule dynamics. During process outgrowth and remodeling a locally reduced microtubule stabilizing activity of tau is required. This can be accomplished by phosphorylation of the KXGS motifs in the repeat region of tau protein by MARK kinases [2], [50] or possibly by axotrophin mediated tau ubiquitination. It is speculated that loss of axotrophin activity may be analogously to prevention of KXGS phosphorylation leading to microtubule superstability and blocking process formation.

Beside tau protein, we identified the catalytic subunit of PP2B and KLC1 as interaction partners for axotrophin RING-variant domain by yeast two-hybrid assay. We did, however, not accomplish the ubiquitination of these proteins by recombinant axotrophin RING-variant domain. Also, tau ubiquitination by E3 ligase Hdm2 failed. This emphasizes the specificity of the ubiquitinating activity of axotrophin towards tau protein. KLC1 interaction with axotrophin in yeast two-hybrid assay was not only confined to the C-terminal portion containing the RING-variant domain (pEG-axoCT) but was also detectable to a lesser extent with the N-terminal axotrophin domain (pEG-axoNT, data not shown). This interaction may result in intracellular transport of axotrophin-containing protein complexes giving rise to the granular and reticular intracellular localization of axoFL and axoN2. In the light of the recent argument of Lazarov et al. [51] that KLC1 tends to exhibit nonspecific binding to other proteins, the significance of KLC1 interaction with axotrophin remains unclear. The strong interaction of axotrophin with both isoforms of PP2B catalytic subunit is intriguing. Axotrophin mRNA upregulation has been found to be associated with transplantation tolerance [19]. Since PP2B is a crucial signaling molecule in regulating adaptive immunity [52] a potential inhibitory effect of axotrophin on PP2B activity may present an immune tolerance regulatory mechanism in addition to LIF receptor downregulation.

Localization of endogenous axotrophin in mouse and human brain shows the same dual cytoplasmic/nuclear localization like overexpressed EGFP-tagged full-length axotrophin in cell lines. We found that the N-terminal portion comprising aa 1–344 targets axotrophin solely to the nucleus. This nuclear localization may be determined by the first RNA-binding stretch aa 76–109. Although NetNES analysis [53] predicts a leucine-rich nuclear export signal within aa 611–617 (LNLEDFD) after the RING-variant domain our analysis shows that a nuclear export signal must be contained further C-terminal within aa 625–704. The lack of this nuclear export signal in the EGFP-fusion protein axoN1 (aa 1–625) leads to a nuclear speckle-like appearance. The localization of axotrophin to nuclear speckles, a site of the pre-mRNA processing and mRNA export [54] and sequence similarity of axotrophin to serine/arginine-rich (SR) proteins argues for a function of axotrophin in RNA maturation and export. The identification of factors controlling the partition between cytoplasmic and nuclear localization of axotrophin and identification of its nuclear interaction partners remains the target of future work.

## 5 Conclusions

In summary, we have shown that tau protein interacts with axotrophin and becomes ubiquitinated by the axotrophin E3 ubiquitin ligase activity. Mono-ubiquitination of tau occurs in the microtubule-binding region and impairs microtubule binding. The dual residency of axotrophin represents different functions in the cytosol and the nucleus. We present a novel tau modification occurring preferentially on four-repeat tau protein which may impact on the pathogenesis of tauopathies.

## Appendix A Supplementary data

Suppl Fig. 1. Multiple sequence alignment of axotrophin. Sequence alignment was done with ClustalX2 (Larkin et al., 2007; Clustal W and Clustal X version 2.0. Bioinformatics, 23:2947–2948). A high degree of sequence homology is found from fish to human in the RING-variant domain aa 552-CRIC---CELC-617 (see asterisk on top of the sequences). Higher amino acid homology in the N-terminal part was also noted in short stretches with basic amino acid residues arginine/lysine such as aa 140–150, 276–300, and 391–411 (numbering according human axotrophin sequence). UniProt accession numbers: Q9H992: E3 ubiquitin-protein ligase MARCH7 *Homo sapiens*, H2QIV6: membrane-associated ring finger (C3HC4) 7 Pan troglodytes, F7E884: MARCH7 *Callithrix jacchus*, G1SEZ6: MARCH7 *Oryctolagus cuniculus*, Q9WV66: E3 ubiquitin-protein ligase MARCH7 *Mus musculus*, F7EQU7: MARCH7 *Monodelphis domestica*, E1C485: MARCH7 *Gallus gallus*, F1RAT0: MARCH7 *Danio rerio*, H3CVR1: MARCH7 *Tetraodon nigroviridis*. Suppl. Fig. 2. Tau protein is preferentially mono-ubiquitinated at several sites by the axotrophin RING-variant domain. Biotinylated human tau isoforms 0N4R and 2N3R were incubated with Mg-ATP, E1, E2 UbcH5c, the axotrophin RING-variant domain in the presence of ubiquitin (lane 1 and lane 2) or methylated ubiquitin (m) blocking ubiquitin chain elongation (lane 3 and lane 4). After separating proteins via SDS-PAGE and immunoblotting using an anti-tau antibody a similar tau protein laddering is observed comparing ubiquitin and methylated ubiquitin. The use of methylated ubiquitin leads only to a reduction of the faint high molecular weight smear (compare lane 1 and lane 3). Suppl. Fig. 3. Co-localization of axotrophin and tau protein in transfected primary neurons. Rat hippocampal neurons were transfected with EGFP-axotrophin and RFP-tau fusion protein expressing plasmids. Fluorescence of expressed proteins was analyzed by confocal laser scanning microscopy. Axotrophin and tau protein are present in the soma and neurites of transfected cells and are co-localized in branching points of neurites (upper inset) and in cytoplasmic structures in the cell body (lower inset). Scale bar: 20 μm.
