# Supplementary material for: Axotrophin/MARCH7 acts as an E3 ubiquitin ligase and ubiquitinates tau protein in vitro impairing microtubule binding
Source: Biochim Biophys Acta Mol Basis Dis. 2014 Sep;1842(9):1527–38. doi: 10.1016/j.bbadis.2014.05.029 (PMC4311138; doi:10.1016/j.bbadis.2014.05.029)
Supplement: Supplementary file 1 — Suppl Fig. 1. Multiple sequence alignment of axotrophin. Sequence alignment was done with ClustalX2 (Larkin et al., 2007; Clustal W and Clustal X version 2.0. Bioinformatics, 23:2947–2948). A high degree of sequence homology is found from fish to human in the RING-variant domain aa 552-CRIC---CELC-617 (see asterisk on top of the sequences). Higher amino acid homology in the N-terminal part was also noted in short stretches with basic amino acid residues arginine/lysine such as aa 140–150, 276–300, and 391–411 (numbering according human axotrophin sequence). UniProt accession numbers: Q9H992: E3 ubiquitin-protein ligase MARCH7 Homo sapiens, H2QIV6: membrane-associated ring finger (C3HC4) 7 Pan troglodytes, F7E884: MARCH7 Callithrix jacchus, G1SEZ6: MARCH7 Oryctolagus cuniculus, Q9WV66: E3 ubiquitin-protein ligase MARCH7 Mus musculus, F7EQU7: MARCH7 Monodelphis domestica, E1C485: MARCH7 Gallus gallus, F1RAT0: MARCH7 Danio rerio, H3CVR1: MARCH7 Tetraodon nigroviridis. Suppl. Fig. 2. Tau protein is preferentially mono-ubiquitinated at several sites by the axotrophin RING-variant domain. Biotinylated human tau isoforms 0N4R and 2N3R were incubated with Mg-ATP, E1, E2 UbcH5c, the axotrophin RING-variant domain in the presence of ubiquitin (lane 1 and lane 2) or methylated ubiquitin (m) blocking ubiquitin chain elongation (lane 3 and lane 4). After separating proteins via SDS-PAGE and immunoblotting using an anti-tau antibody a similar tau protein laddering is observed comparing ubiquitin and methylated ubiquitin. The use of methylated ubiquitin leads only to a reduction of the faint high molecular weight smear (compare lane 1 and lane 3). Suppl. Fig. 3. Co-localization of axotrophin and tau protein in transfected primary neurons. Rat hippocampal neurons were transfected with EGFP-axotrophin and RFP-tau fusion protein expressing plasmids. Fluorescence of expressed proteins was analyzed by confocal laser scanning microscopy. Axotrophin and tau protein are present in the som [file mmc1.pdf]

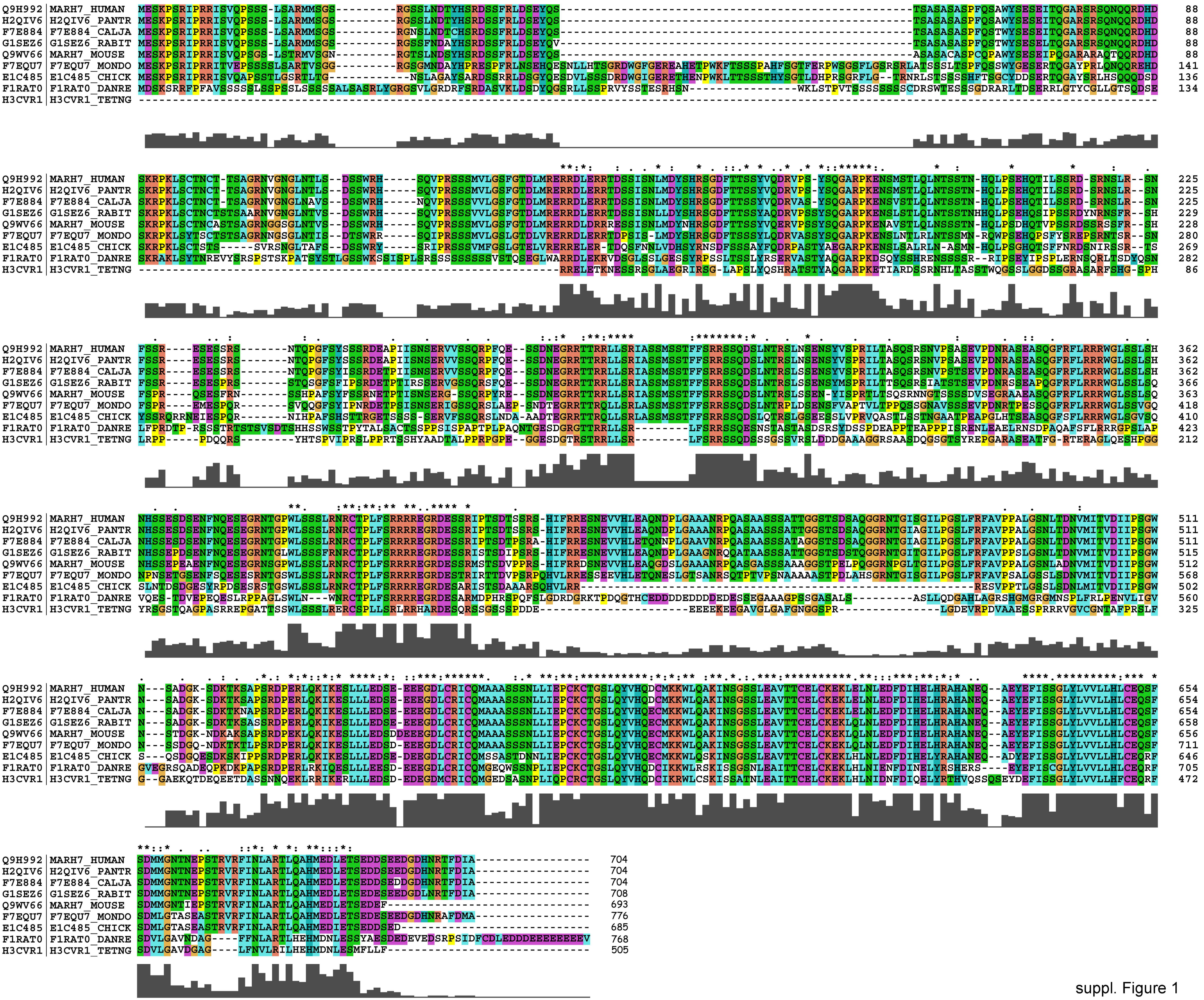


suppl. Figure 1

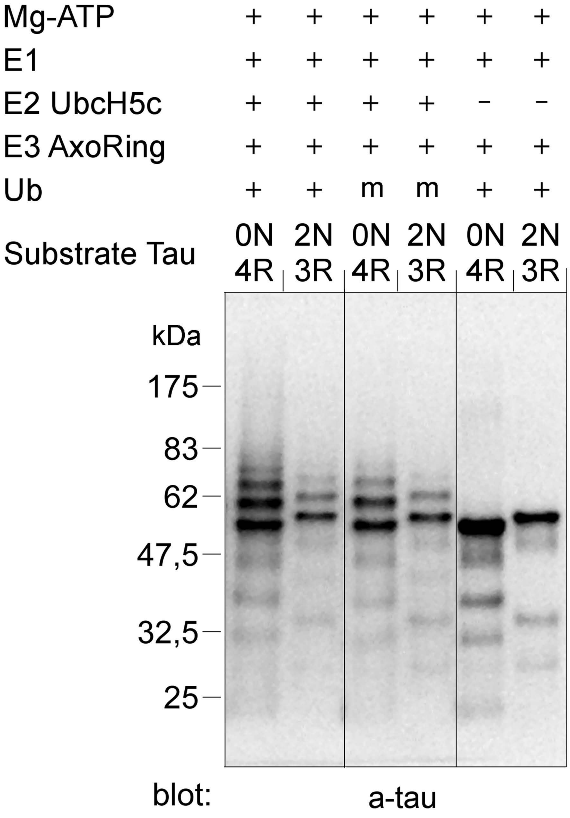

Mg-ATP + + + + + +
E1 + + + + + +
E2 UbcH5c + + + + – –
E3 AxoRing + + + + + +
Ub + + m m + +
Substrate Tau 0N 4R 2N 3R 0N 4R 2N 3R 0N 4R 2N 3R
kDa
175
83
62
47,5
32,5
25
blot: a-tau


suppl. Fig. 2

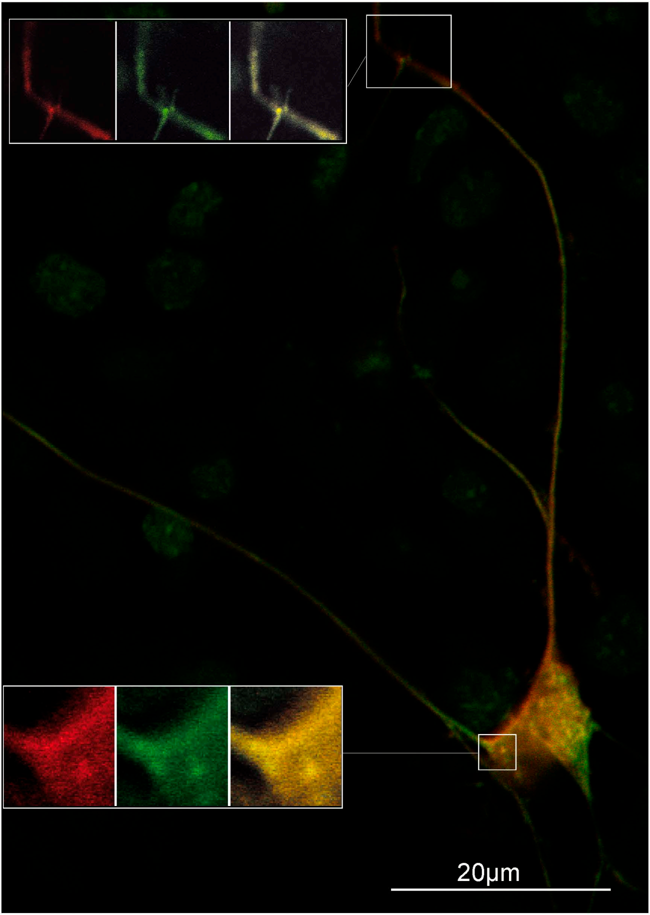


suppl. Figure 3
