# Supplementary material for: Axotrophin/MARCH7 acts as an E3 ubiquitin ligase and ubiquitinates tau protein in vitro impairing microtubule binding
Source: Biochim Biophys Acta Mol Basis Dis. 2014 Sep;1842(9):1527–38. doi: 10.1016/j.bbadis.2014.05.029 (PMC4311138; doi:10.1016/j.bbadis.2014.05.029)
Supplement: Supplementary file 2 — Supplementary tables. [file mmc2.doc]

**SupplementaL TaBLE**

Table I Summary of plasmids used in yeast two-hybrid interaction assay

| plasmid | vector | site | protein acc. | amino acids | remark |
| --- | --- | --- | --- | --- | --- |
| pEG-axo | pEG202NLS | BamHI/SalI | NP_073737 | 1-704 | autoactivation |
| pEG-axoNT | pEG202NLS | BamHI/SalI | NP_073737 | 1-538 |  |
| pEG-axoCT | pEG202NLS | BamHI/SalI | NP_073737 | 552-682 |  |
| pEG-tau | pEG202 | BamHI/XhoI | NP_005901 | 1-441 | Co-IP |
| pBait | pEG202 | EcoRI/SalI | NP_036907  TGFBR1 | 149-346 | pos. control with pTarget |
|  | Vector | site | protein acc. | amino acids |  |
| pJG-tau | pJG4-5 | EcoRI/XhoI | NP_005901 | 1-441 |  |
| pJG-axo | pJG4-5 | EcoRI/XhoI | NP_073737 | 1-704 | Co-IP |
| pJG-neg | pJG4-5 | EcoRI/XhoI | NP_115786  Hook3 | 1-718 | neg. control |
| pJG-PP2Bα | pJG4-5 | EcoRI/XhoI | NP_000935 | 1-521 |  |
| pJG-PP2Bβ | pJG4-5 | EcoRI/XhoI | NP_066955 | 1-524 |  |
| pJG-UbcH5a | pJG4-5 | EcoRI/XhoI | NP_003329 | 1-147 |  |
| pJG-UbcH5c | pJG4-5 | EcoRI/XhoI | NP_003331 | 1-147 |  |
| pJG-UbcH12 | pJG4-5 | EcoRI/XhoI | NP_003960 | 1-183 |  |
| pJG-UbcH13 | pJG4-5 | EcoRI/XhoI | NP_003339 | 1-152 |  |
| pJG-KLC | pJG4-5 | EcoRI/XhoI | NP_005543 | 1-560 |  |
| pTarget | pJG4-5 | EcoRI/XhoI. | AAI26072  FKBP-12 | 1-108 | pos. control with pBait |

Table II Profile of autoptic brain tissue donors

| case | sex | age | cause of death | post-mortem interval | diagnosis |
| --- | --- | --- | --- | --- | --- |
|  |  |  |  |  |  |
| 1 | F | 79 | kidney failure | 33 | control |
| 2 | M | 64 | cardiovascular failure | 30 | control |
| 3 | M | 66 | hemorrhagic shock | 24 | control |
| 4 | M | 73 | cardiovascular failure | 24 | control |
| 5 | F | 84 | cardiovascular failure | 36 | control |
| 6 | M | 82 | cardiovascular failure | 46 | control |
| 7 | M | 76 | cardiovascular failure | 46 | control |
| 8 | F | 86 | cardiac arrest | 40 | control |
| 9 | F | 84 | cardiovascular failure | 12 | control |
| 10 | M | 85 | cardiovascular failure | 48 | control |
| 11 | F | 73 | cardiovascular failure | 34 | AD |
| 12 | F | 60 | pneumonia | 26 | AD |
| 13 | F | 81 | cardiovascular failure | 58 | AD |
| 14 | M | 75 | pneumonia | 48 | AD |
| 15 | M | 84 | liver failure | 29 | AD |
| 16 | F | 86 | pulmonary embolism | 54 | AD |
| 17 | F | 82 | diabetes | 30 | AD |
| 18 | F | 83 | pneumonia | 72 | AD |
| 19 | F | 77 | gastrointestinal bleeding | 34 | AD |
| 20 | M | 75 | pneumonia | 48 | AD |
| 21 | F | 88 | pulmonary embolism | 48 | AD |
| 22 | F | 82 | cardiovascular failure | 24 | AD |
| 23 | F | 82 | pneumonia | 48 | AD |
| 24 | F | 92 | pneumonia | 48 | AD |
